# Low-grade oncocytic fumarate hydratase-deficient renal cell carcinoma in a pediatric liver transplant recipient: a case report

**DOI:** 10.1016/j.eucr.2026.103558

**Published:** 2026-07-27

**Authors:** Abdurrahman İnkaya, Hasan Samet Güngör

**Affiliations:** Department of Urology, Health Sciences University, Umraniye Training and Research Hospital, Istanbul, Turkey

**Keywords:** Fumarate hydratase, Renal cell carcinoma, Pediatric, Nephron-sparing surgery, Liver transplantation, Immunosuppression, Variant of uncertain significance

## Abstract

Fumarate hydratase (FH)-deficient renal cell carcinoma (RCC) is a rare molecularly defined neoplasm, reported only occasionally in children. We describe a 9-year-old liver transplant recipient with prior lymphoma, chronic immunosuppression, hypertension, and reduced renal reserve who presented with a complex cystic left upper-pole renal mass. Open partial nephrectomy was performed using local parenchymal compression without hilar clamping to avoid global ischemia. Histopathology confirmed low-grade oncocytic FH-deficient RCC (pT1a, R0), and renal function remained stable. Germline FH sequencing identified a variant of uncertain significance, warranting genetic counseling, variant reinterpretation, and long-term surveillance.

## 1 Introduction

Renal cell carcinoma is uncommon in childhood and differs substantially from its adult counterpart, with translocation-associated tumors and other molecularly defined entities predominating in pediatric and adolescent cases.^1^^,^^2^ Fumarate hydratase (FH)-deficient RCC is recognized for its association with FH tumor predisposition syndrome, previously termed hereditary leiomyomatosis and renal cell carcinoma syndrome.^3^^,^^4^ Most reported adult cases present at an advanced stage with aggressive behavior,^3^ although a low-grade oncocytic morphological variant has been described that can resemble succinate dehydrogenase (SDH)-deficient RCC and other low-grade oncocytic renal neoplasms.^5^^,^^6^ Immunohistochemical loss of FH expression, together with positive 2-succinocysteine (2SC) staining, supports the diagnosis, whereas retained SDHB expression helps exclude SDH-deficient RCC.^5^^,^^7^ Germline testing is necessary to establish whether an inherited predisposition syndrome is present.^4^ We report a pediatric case of low-grade oncocytic FH-deficient RCC arising in a liver transplant recipient with chronic immunosuppression, prior lymphoma, and impaired renal reserve, managed with open nephron-sparing surgery without global renal ischemia.

## 2 Case presentation

A 9-year-old girl was referred for evaluation of a newly detected left renal mass. Her medical history included biliary atresia treated surgically in infancy, followed by maternal living-donor liver transplantation, for which she received tacrolimus-based immunosuppression and tenofovir for chronic hepatitis B infection. She had previously been treated for Burkitt lymphoma with renal involvement complicated by temporary dialysis, and subsequently for recurrent Hodgkin lymphoma with vertebral and intracranial involvement. Long-term sequelae included hypertension and chronic renal parenchymal disease.

**Fig. 1:**
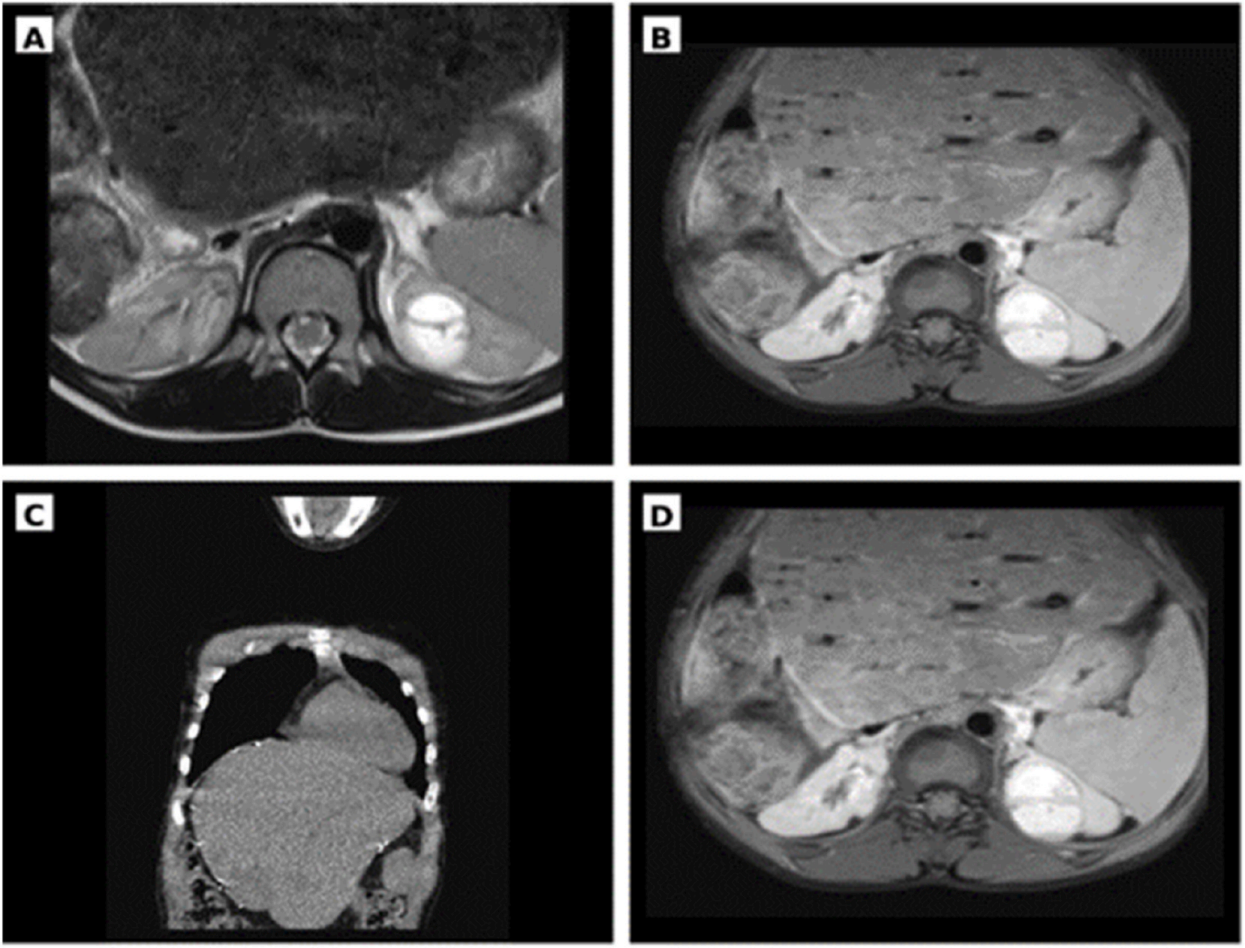
Preoperative cross-sectional imaging. (A) Axial T2-weighted MRI shows a well-circumscribed, exophytic, predominantly cystic lesion arising from the upper pole of the left kidney with internal septation. (B) Axial contrast-enhanced MRI demonstrates complex cystic morphology with enhancing septal components. (C) Coronal CT provides an overview of the upper abdominal postoperative/transplant-related anatomy. (D) Axial contrast-enhanced MRI demonstrates enhancing solid components within the cystic lesion.

The renal lesion was identified during routine nephrology follow-up. Magnetic resonance imaging revealed an exophytic, septated, complex cystic lesion in the upper pole of the left kidney measuring approximately 40× 30 mm, hyperintense on T1-and T2-weighted sequences, with diffusion-restricting components and septal enhancement after contrast administration (Fig. 1). No lymphadenopathy was identified. Ultrasonography revealed a 35 × 34 mm heterogeneous hypoechoic solid lesion with hyperechoic nodular and fibrotic areas. Given the patient's history of renal lymphomatous involvement, prior chemotherapy, and chronic renal injury, the differential diagnosis included complex renal cyst, cystic RCC, lymphomatous renal involvement, post-treatment change, and rare molecularly defined renal neoplasms.

Dimercaptosuccinic acid renal scintigraphy demonstrated globally reduced bilateral cortical uptake, with differential renal function of 60% in the left kidney and 40% in the right kidney. Preoperative serum creatinine was 0.84 mg/dL, hematocrit was 43%, urine culture was sterile, and hepatitis B surface antigen was positive. Pediatric gastroenterology was consulted regarding perioperative tacrolimus management, and cardiology evaluation, including echocardiography, revealed no contraindication to surgery. There was no family history of renal cancer, cutaneous or uterine leiomyomas, or hereditary cancer syndromes.

Because bilateral cortical function was globally reduced, the contralateral kidney was smaller, and the patient had hypertension and a prior history of dialysis, radical nephrectomy was avoided in favor of open partial nephrectomy. Under general anesthesia, with the patient in the right lateral decubitus position, the left kidney was approached retroperitoneally through a flank incision without entering the peritoneal cavity. After mobilization within Gerota fascia, a 4-cm upper-pole lesion was identified. Hilar clamping was not performed; instead, local upper-pole parenchymal compression with atraumatic bowel clamps provided regional vascular control while avoiding global renal ischemia. The tumor margin was marked with electrocautery, and enucleoresection was performed. Excision and renorrhaphy were completed within 11 minutes of the onset of compression. Renorrhaphy used one V-Loc and four CT-1 sutures supplemented with fibrillar hemostatic material. No active bleeding was observed after release of compression, and no intraoperative complications occurred.

The partial nephrectomy specimen measured 4.5 x 4 × 3.5 cm and contained a biloculated cystic area with hemorrhagic serous fluid. An irregular, dirty-white tumor measuring 3.5 x 2 × 2 cm was identified within the cyst wall, partly surrounded by a fibrotic band. Although the tumor approached the parenchymal margin in some areas, all final surgical margins were negative. Microscopically, the tumor displayed oncocytic morphology with acinar, tubular, nodular, and microcystic growth patterns, without sarcomatoid differentiation, perirenal or sinus fat invasion, vascular invasion, collecting system invasion, or perineural invasion. Immunohistochemistry showed diffuse PAX8 and EMA positivity, AMACR and GATA3 positivity, and retained SDHB expression; CK7, CD10, CD117, CA9, WT1, cathepsin K, CK20, Melan-A, HMB45, and TFE3 were negative, and TFEB showed cytoplasmic positivity. Nuclear FH expression was lost, and 2SC staining was positive, supporting a diagnosis of low-grade oncocytic FH-deficient RCC, staged pT1a, Nx, R0, V0, L0 (AJCC 8th edition). The non-neoplastic parenchyma showed periglomerular fibrosis, mesangial expansion, segmental sclerosis, and tubular atrophy with interstitial fibrosis, consistent with chronic renal injury.

Serum creatinine was 0.91 mg/dL on postoperative day 1; the drain was removed the same day because no output was recorded, and the patient was discharged on postoperative day 2. On postoperative day 6, she presented with abdominal distension and colicky pain; she remained clinically stable, tolerated oral intake, and had no vomiting. Ultrasonography showed no free intra-abdominal fluid, and symptoms resolved after an enema. At 1 month, serum creatinine was 0.88 mg/dL, and at 3 months, imaging showed postoperative changes without local recurrence or metastatic disease (Table 1). Subsequent germline FH sequencing identified an FH variant classified as a variant of uncertain significance (VUS). Because the clinical significance of this variant has not been established, the result was not interpreted as confirmation of FH tumor predisposition syndrome. Genetic counseling was provided, and periodic reassessment of the variant classification was recommended.

**Table 1:** Clinical timeline.

Time point	Clinical event
Infancy	Surgery for biliary atresia followed by liver transplantation.
Childhood	Burkitt lymphoma with reported renal involvement, treated with chemotherapy; temporary dialysis.
Later childhood	Recurrent Hodgkin lymphoma with vertebral and intracranial involvement and additional chemotherapy.
Month 1	MRI revealed a new complex septated left upper-pole renal lesion (∼4 cm).
Month 3	DMSA scintigraphy revealed reduced bilateral cortical uptake, with differential function of 40% on the right and 60% on the left.
Day 0	Open left partial nephrectomy/enucleoresection with local parenchymal compression and no global ischemia.
Postoperative day 1	Serum creatinine was 0.91 mg/dL, and the drain was removed because no output was recorded.
Postoperative day 2	Discharge.
1 month	Serum creatinine was 0.88 mg/dL.
3 months	Imaging showed postoperative changes without local recurrence or metastatic disease.
Follow-up plan	Germline FH sequencing identified a variant of uncertain significance; genetic counseling and periodic variant reinterpretation were recommended.
Ongoing	Long-term MRI-based renal surveillance is planned.

## 3 Discussion

This case illustrates several features that complicate the diagnosis and management of pediatric renal masses. Molecularly defined renal epithelial tumors remain incompletely characterized in children,^1^^,^^8^ and in this patient the clinical picture was further obscured by prior lymphomatous renal involvement, chemotherapy, chronic immunosuppression, and pre-existing renal impairment. Radiologically, the complex cystic and solid appearance of the lesion, with septations and enhancing components, raised concern for lymphoma-related change as well as a primary renal neoplasm; new development and contrast enhancement ultimately favored surgical excision over surveillance.

Renal preservation was the principal determinant of the surgical strategy. Scintigraphy confirmed that the left kidney, harboring the tumor, contributed a larger share of total renal function, so radical nephrectomy would have carried a substantial functional cost. Open nephron-sparing surgery using local parenchymal compression achieved complete excision while avoiding hilar clamping and global ischemia. Early postoperative creatinine values support the short-term functional adequacy of this approach, although longer follow-up is needed to confirm its durability.

Pathological diagnosis depended on integrating morphology with immunohistochemistry, because low-grade oncocytic FH-deficient RCC can resemble SDH-deficient RCC and other oncocytic renal tumors.^5^, ^6^, ^7^ Retained SDHB expression argued against an SDH-deficient tumor, whereas loss of nuclear FH expression and positive 2SC staining supported FH deficiency. Organ-confined stage, negative margins, and absence of lymphovascular invasion or sarcomatoid differentiation were favorable features.

Germline testing in this case identified an FH variant of uncertain significance. A negative family history does not eliminate the possibility of a hereditary predisposition, because penetrance may be variable, familial assessment may be incomplete, and de novo events may occur.^4^^,^^9^ A VUS is a sequence alteration for which available evidence is insufficient to determine whether it is disease-causing or benign.^10^ Therefore, this finding neither confirms nor excludes FH tumor predisposition syndrome. The tumor diagnosis remains supported by morphology and immunohistochemistry, including FH loss and 2SC positivity, but the germline result should be interpreted as unresolved rather than pathogenic. Conversely, a VUS should not be used for predictive testing or definitive risk assignment in relatives unless it is reclassified. Ongoing genetic counseling and periodic variant reinterpretation according to updated classification frameworks are therefore essential.

Although early oncological follow-up was reassuring, the interval remains short. Given the aggressive behavior reported for conventional FH-deficient RCC, low-grade oncocytic morphology should not justify reduced surveillance in a child until further pediatric outcome data are available.^3^^,^^6^ MRI-based surveillance is preferable to limit cumulative radiation exposure.

This report is limited by the short follow-up interval, which precludes firm conclusions regarding long-term oncological behavior. The lack of tumor sequencing also constrains molecular characterization and prevents direct comparison between the tumor-level FH-deficient phenotype and the germline VUS. Nevertheless, the case highlights the diagnostic and operative challenges posed by a rare FH-deficient renal tumor in a child with complex comorbidities and supports individualized nephron-sparing surgery when complete excision is achievable and renal preservation is clinically critical.

## 4 Conclusions

Low-grade oncocytic FH-deficient RCC can occur in childhood and may present as a complex cystic renal mass. In this patient, open nephron-sparing surgery with local parenchymal compression achieved complete tumor excision without global renal ischemia while preserving early renal function. Loss of FH expression and 2SC positivity warrant germline FH testing irrespective of family history. When germline testing identifies an FH VUS, the result should be interpreted cautiously and should not be regarded as diagnostic of FH tumor predisposition syndrome unless reclassified. Continued genetic counseling, periodic variant reinterpretation, and long-term MRI-based surveillance are warranted given the incompletely defined oncological behavior of pediatric FH-deficient RCC.

## Patient consent

Written informed consent was obtained from the patient's legal guardian for publication of this case report and accompanying anonymized clinical images. All identifying information was removed, and the report was prepared in accordance with the CARE case-report guidance.

## Funding

This research did not receive any specific grants from funding agencies in the public, commercial, or not-for-profit sectors.

## CRediT authorship contribution statement

**Abdurrahman İnkaya:** Conceptualization, Data curation, Formal analysis, Investigation, Methodology, Project administration, Resources, Supervision, Validation, Writing – original draft, Writing – review & editing. **Hasan Samet Güngör:** Conceptualization, Data curation, Formal analysis, Investigation, Methodology, Project administration, Resources, Supervision, Validation, Visualization, Writing – original draft, Writing – review & editing.

## Conflict of interest

The authors declare no conflict of interest.
